# Plasticity within excitatory and inhibitory pathways of the vestibulo-spinal circuitry guides changes in motor performance

**DOI:** 10.1038/s41598-017-00956-5

**Published:** 2017-04-12

**Authors:** Diana E. Mitchell, Charles C. Della Santina, Kathleen E. Cullen

**Affiliations:** 1 Department of Physiology McGill University, Montreal, QC Canada; 2 Department of Otolaryngology - Head & Neck Surgery Johns Hopkins University School of Medicine, Baltimore, MD USA

## Abstract

Investigations of behaviors with well-characterized circuitry are required to understand how the brain learns new motor skills and ensures existing behaviors remain appropriately calibrated over time. Accordingly, here we recorded from neurons within different sites of the vestibulo-spinal circuitry of behaving macaque monkeys during temporally precise activation of vestibular afferents. Behaviorally relevant patterns of vestibular nerve activation generated a rapid and substantial decrease in the monosynaptic responses recorded at the first central stage of processing from neurons receiving direct input from vestibular afferents within minutes, as well as a decrease in the compensatory reflex response that lasted up to 8 hours. In contrast, afferent responses to this same stimulation remained constant, indicating that plasticity was not induced at the level of the periphery but rather at the afferent-central neuron synapse. Strikingly, the responses of neurons within indirect brainstem pathways also remained constant, even though the efficacy of their central input was significantly reduced. Taken together, our results show that rapid plasticity at the first central stage of vestibulo-spinal pathways can guide changes in motor performance, and that complementary plasticity on the same millisecond time scale within inhibitory vestibular nuclei networks contributes to ensuring a relatively robust behavioral output.

## Introduction

**Figure 1:**
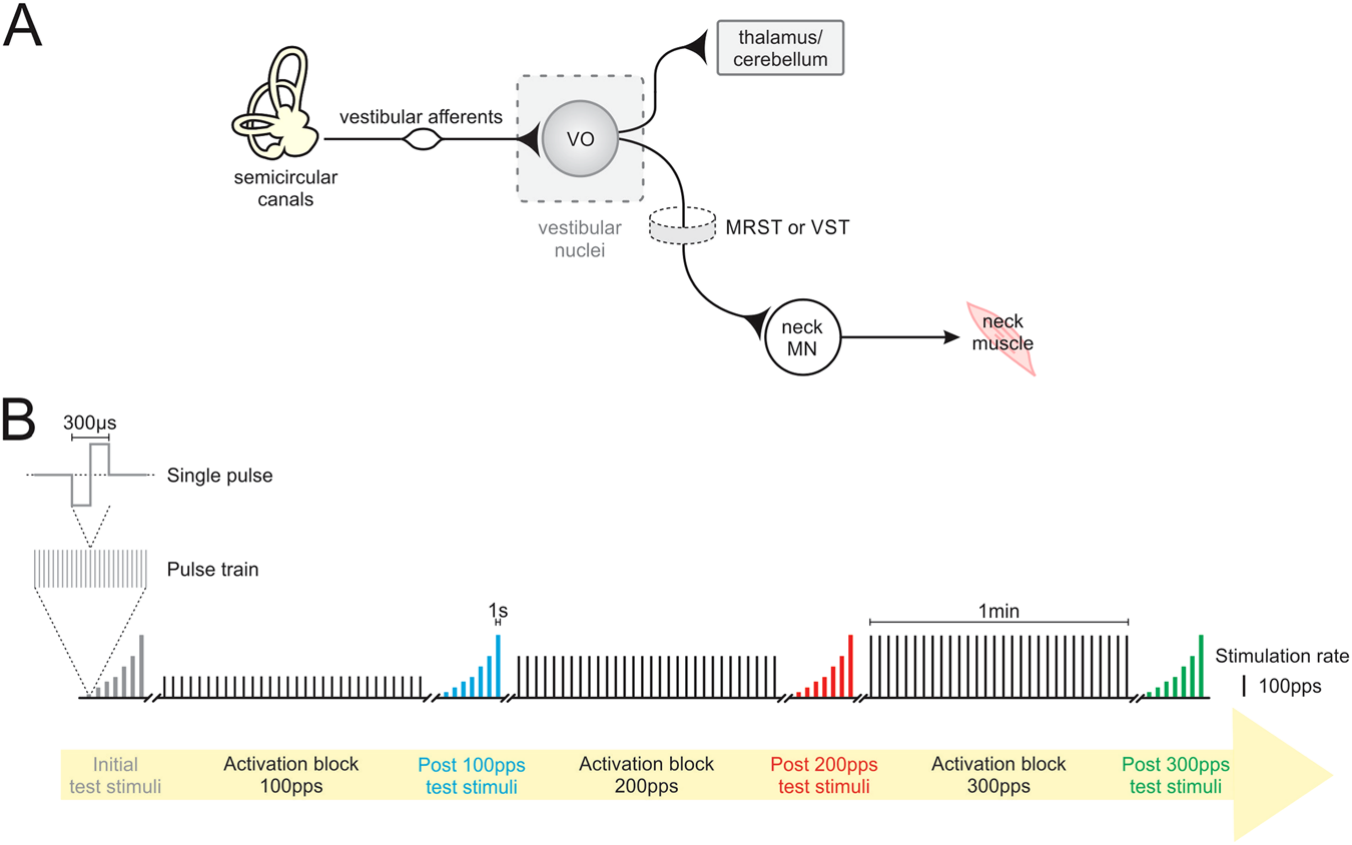
Vestibulo-collic reflex pathway and stimulation protocol. (**A**) Diagram of pathways mediating the vestibulo-collic reflex. MN, motoneurons; MRST, medial reticulospinal tract; VST, vestibulospinal tract. (**B**) Timeline of stimulation protocol. Each rectangle represents a pulse train in which the width represents the duration and the height represents the pulse rate. Test stimuli consisted of pulse trains lasting 1 s delivered at rates of 25–300pps. Activation stimuli consisted of 30 pulse trains lasting 500 ms delivered every 2 s at pulse rates of 100, 200 and 300pps.

Rapid reflexive postural adjustments ensure the maintenance of stable vision and upright stance with respect to our surroundings during everyday activities. The vestibular system plays a fundamental role in the generation of these reflexes, which are mediated by well-defined neural circuitry (reviewed in ref. 1). For example, one particularly well studied vestibulo-spinal reflex is the vestibulo-collic reflex, which serves to stabilize the head in space (reviewed in ref. 2). The efficacy of the pathway mediating this reflex is evident when the vestibular nerve is electrically stimulated; compensatory head movements are consistently evoked in the direction required to stabilize the head to support postural stability^3–6^. The direct vestibulo-collic reflex pathway is mediated by a distinct subset of neurons within the vestibular nuclei, termed vestibular-only (VO) neurons, that receive input from the vestibular nerve and in turn transmit this sensory information to neck motoneurons via descending projections through the spinal cord (Fig. 1A)^7–10^.

While the ability to generate vestibulo-spinal reflexes is essential for survival, it is not sufficient. The motor output of these reflexes must be rapidly tailored to meet new constraints imposed by changes in the external environment and/or biomechanical dynamics (e.g., changes in muscle state). Indeed, accumulating evidence suggests that vestibulo-spinal reflexes rapidly adapt to changes in sensory input. For instance, while patients with peripheral vestibular damage initially experience postural deficits (e.g., head tilt towards the lesioned side; reviewed in refs 11 and 12), these symptoms commonly subside within the first week. Similarly after prolonged exposure to microgravity, astronauts experience substantial impairments in posture and locomotion upon returning to earth^13–15^, which then recover following a comparable time course (reviewed in ref. 16). Furthermore, repeated trials of applied linear acceleration stimulation reduce the activation of neck muscle activation suggesting that the efficacy of vestibular-spinal pathways can be modulated over even a shorter time window by previous experience^17–19^.

The prevailing view is that sensorimotor learning in vestibulo-spinal reflex pathways is initially driven by adaptive changes in the vestibulo-cerebellum. Notably, lesions of the anterior vermis impair adaptive modifications of vestibulo-spinal reflexes^20, 21^, while plasticity within the brainstem (e.g., vestibular nuclei) occurs over longer time scales of hours to days^22^. Further a recent report has shown that cerebellar-dependent mechanisms contribute to the induction of homeostatic plasticity in order to maintain an optimal working range of the vestibulo-ocular reflex^23^. On the other hand, *in vitro* studies have shown that repetitive stimulation of the vestibular nerve can alter the strength of afferent synapses onto the vestibular nuclei neurons over a time scale of minutes^24, 25^ (Fig. 1B). Although such rapid synaptic plasticity within brainstem pathways would be expected to fine-tune the performance of vestibulo-spinal reflexes, to date no study has directly tested this hypothesis *in vivo*.

Accordingly, here we directly tested whether plasticity occurs at the first central synapse of vestibulo-spinal pathways *in vivo* and whether this plasticity in turn modifies motor performance in awake behaving monkeys. Single unit recordings were made from central neurons that receive direct input from vestibular nerve afferents. In response to behaviorally relevant patterns of vestibular nerve activation at rates spanning the physiological range of afferent firing rates (i.e., 300–400 sp/s^26^), we found that the probability of evoking a spike was significantly attenuated on a monosynaptic timescale. In contrast, the probability of evoking a spike from the vestibular afferents that provide input to these neurons remained constant, suggesting that plasticity was induced at the vestibular afferent-central neuron synapse. To investigate the impact of this plasticity on motor performance, we quantified the recorded head movements and found a significant decrease in the vestibulo-spinal reflex response, which lasted up to 8 hours. Notably, the relative decrease in motor response was significantly less than that of the attenuation observed at the afferent-central neuron synapse. We thus further determined whether changes in local brainstem pathways might compensate for the decreased efficacy of the vestibular afferent-central neuron synapse. Consistent with this proposal, we found that the decreased probability of evoking spiking within neurons in local inhibitory pathways, which occurred on a polysynaptic timescale, remained robust and unchanged following vestibular nerve activation. Overall, our results link rapid plasticity at the first central synapse of the vestibulo-spinal circuitry to changes in motor performance and suggest that complementary plasticity within inhibitory vestibular nuclei pathways contributes to ensuring relatively constant behavioral output.

## Results

Plasticity within the vestibulo-spinal circuitry is required to fine-tune sensory-motor performance to ensure the maintenance of accurate posture. In order to understand how synaptic plasticity leads to changes in neuronal activity to shape behavioral performance, we studied whether behaviorally relevant patterns of vestibular nerve activation can induce changes in the probability of firing in vestibular nuclei neurons. We first addressed whether and how plasticity occurs at the first central synapse within vestibulo-spinal pathways by recording the activity of neurons known to receive direct input from the vestibular nerve, and in turn, project to the cervical spinal cord. We next assessed the impact of this plasticity on the efficacy of vestibulo-spinal reflexes by recording the compensatory head motion that is driven by activation of this circuitry. We then investigated whether there were more global changes at the level of the vestibulo-spinal network by recording the activity of neurons that receive indirect inhibitory input from the stimulated nerve. Finally, we determined the time course over which neuronal and behavioral responses returned to baseline levels following vestibular nerve activation, and tested whether stimulation of pathways with natural motion expedites recovery.

### Nerve activation induced plasticity in direct vestibulo-spinal pathway neurons

**Figure 2:**
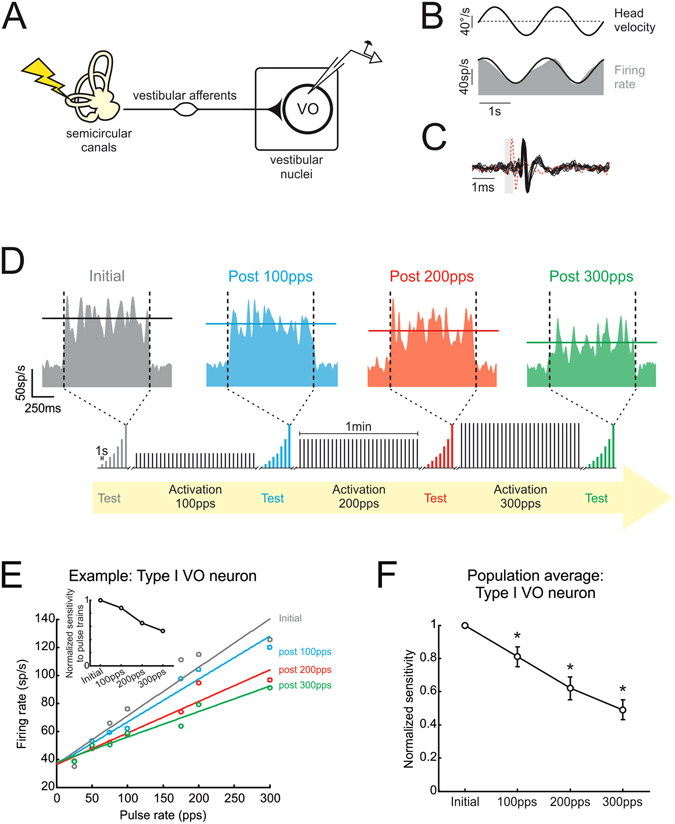
Type I VO neurons are attenuated following activation of the vestibular nerve. (**A**) Recordings were made from VO neurons receiving direct input from the vestibular nerve during stimulation of the horizontal semicircular canal. (**B,C**) Example type I VO neuron increased its firing as a function of ipsilateral head motion and reliably fired action potentials in response to individual pulses. The red trace shows a trial during which the neuron failed to fire an action potential because the pulse occurred during its refractory period. (**D**) Response of example type I VO neuron during pulse trains delivered at 300pps during test stimuli before and after activation of the vestibular nerve. Inset below shows timeline of stimulation protocol. (**E**) Firing rate as a function of pulse rate measured during test stimuli applied before and after activation of the vestibular nerve. Inset shows slope of linear regression between firing rate and pulse rate before and after activation of the vestibular nerve for example neuron. (**F**) Normalized sensitivity to test stimuli for population of type I VO neurons is attenuated following activation of the vestibular nerve. Error bars represent ± s.e.m.

We began by first establishing whether plasticity could be induced in awake, behaving primates at the synapse of vestibular afferents onto a class of neurons in the vestibular nuclei termed vestibular-only (VO) neurons (Fig. 2A). In particular, type I VO neurons receive direct input from the vestibular nerve and project bilaterally to the spinal cord^7, 9, 10, 27^, and via their projections to the spinal cord contribute to vestibulo-spinal reflexes (reviewed in refs 7 and 27). We identified VO neurons (N = 22) based on three key criteria, consistent with previous characterizations^28–30^. First, as is illustrated for a typical cell in Fig. 2B, neuronal firing rates increased in response to ipsilateral head motion (i.e., a type I response) during passively applied whole-body rotation [0.63 ± 0.09 (sp/s)/(°/s), 0.5 Hz ± 40°/s], and led head velocity (17 ± 3°). Second, each neuron was unresponsive to eye movements, including saccades, sustained changes in ocular fixation angle as well as smooth pursuit when the head was stationary. Finally, as illustrated in Fig. 2C for the same example neuron, vestibular nerve activation induced action potentials at monosynaptic latencies (0.7–1.3 ms) in all type I VO neurons, consistent with their direct input from the ipsilateral vestibular nerve.

Once this initial characterization was complete, we tested whether behaviorally relevant patterns of vestibular nerve activation induced changes in the probability of type I VO neuron firing. Recordings were made in complete darkness from the same neurons before and after the application of a stimulation protocol previously shown to induce plasticity at the first central vestibular synapse *in vitro* (Fig. 2D lower inset)^24, 25^, and because the pulse rates fall within the physiological range of vestibular afferent firing rates^26^. Specifically, we first applied a short test sequence, consisting of pulse trains lasting 1 s, delivered at constant rates ranging from 25–300pps, to determine the baseline sensitivity of each neuron to different stimulation rates. We then applied 3 blocks of activation each lasting 1 minute, and comprising of 30 pulse trains at either 100, 200 or 300pps (Fig. 1B). To establish whether this sustained stimulation had altered the efficacy of vestibular afferent synapses onto type I VO neurons, we then recorded neuronal activity once more during the short test sequence. Figure 2D shows the activity of our example neuron in response to stimulation at 300pps within each of these test sequences. Initially, the firing rate of the example neuron exhibited a robust increase at the onset of the pulse train (average firing rate for example neuron = 126 ± 0.9 sp/s; Fig. 2D, gray panel). However, following the first activation block at 100pps, this neuron still responded to the 300pps test stimulation but its modulation was reduced (average firing rate for example neuron = 118 ± 0.8 sp/s; Fig. 2D, blue panel). Moreover, in response to each of the two subsequent activation blocks neuronal modulation further decreased (average firing rate for example neuron = 104 ± 0.9 and 82 ± 0.8 sp/s, respectively; red and green panels in Fig. 2D).

To quantify this decreased modulation, we measured the neuronal activity induced over the full range of pulse rates delivered during each short test sequence (25–300pps). The slope of the regression between the firing rate and pulse rate served as a measure of the neuron’s sensitivity to the test stimuli. Initially, the firing rate of the example neuron increased linearly with pulse rate (gray data points in Fig. 2E), similar to what has been observed *in vitro* ^31^. Following activation at 100pps, while the relationship between pulse rate and firing rate remained linear, the neuron’s sensitivity was reduced (i.e., the slope of the regression between pulse rate and firing rate decreased; blue data points in Fig. 2E). Furthermore, following each of the two subsequent activation blocks (i.e., 200 and 300pps stimulation), the neuron became progressively less sensitive to the test stimuli (red and green data points in Fig. 2E, respectively). We quantified this across our population by computing the slope of the linear relationship between firing rate and pulse rate over each of the four test stimuli, as is illustrated for the example neuron in the inset in Fig. 2E. Overall, the sensitivity of type I VO neurons to the test stimuli significantly decreased following each activation block (Fig. 2F, P < 0.001). In contrast, neuronal resting rates and discharge regularity were unaffected (P > 0.05). Taken together, these results show that behaviorally-relevant patterns of vestibular nerve activation can rapidly induce changes in the firing of type I VO neurons.

### Time course of attenuation in the direct vestibulo-spinal pathway

**Figure 3:**
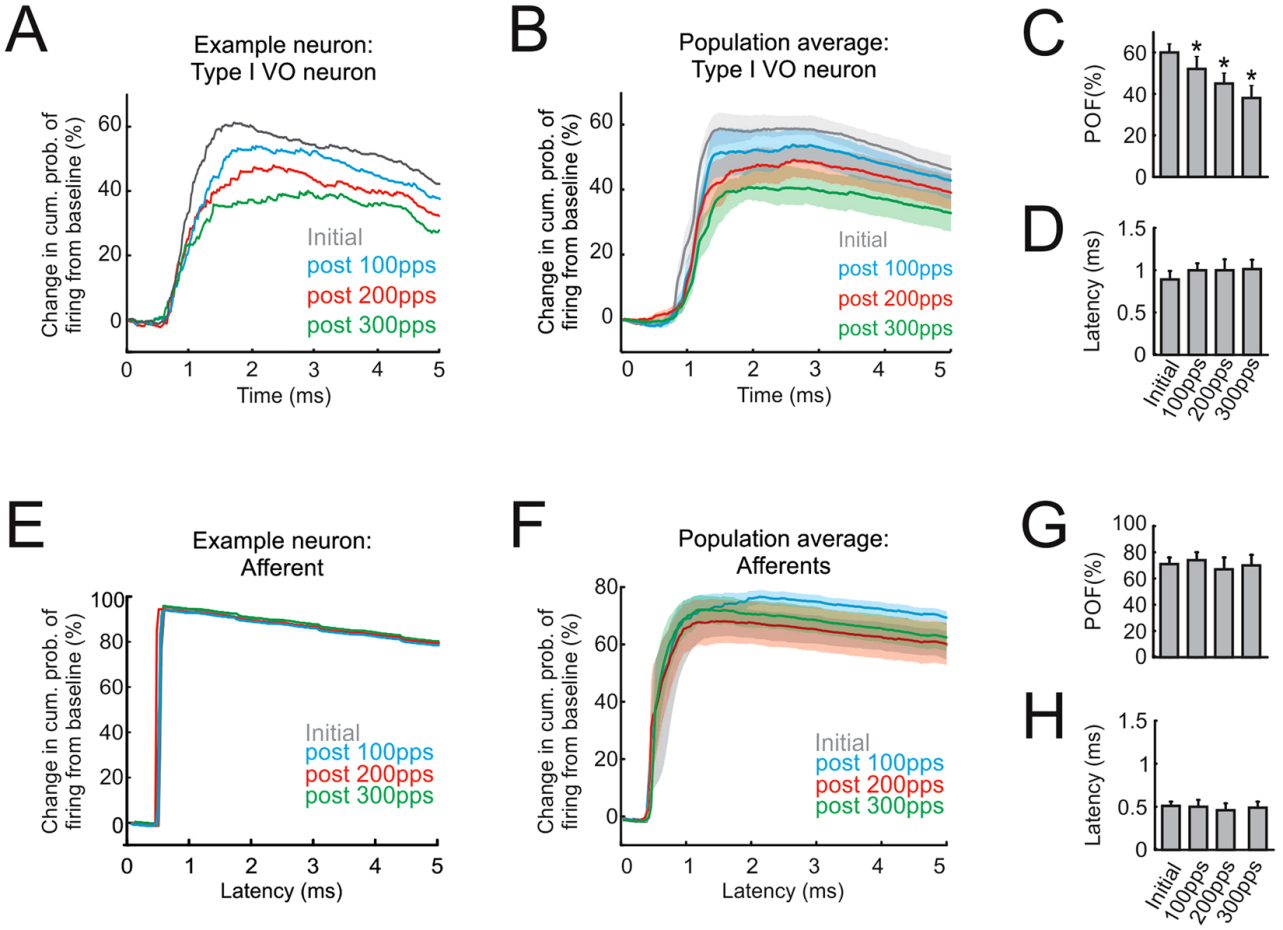
Modulation of vestibular afferent firing remains robust following activation. (**A,B**) Change in cumulative probability of firing from baseline for example (**A**) and population (**B**) of type I VO neurons before and after activation of the vestibular nerve. (**C,D**) Probability of firing (**C**) and latency (**D**) calculated from the change in probability of firing from baseline curves for type I VO neurons. (**E,F**) Change in cumulative probability of firing from baseline for example (**E**) and population (**F**) of vestibular afferents before and after activation of the vestibular nerve. (**G,H**), Probability of firing (**G**) and latency (**H**) calculated from the change in probability of firing from baseline curves for vestibular afferents. Shaded area represent ±s.e.m.

To understand the mechanisms of the plasticity described above, we next evaluated the time scale over which response attenuation occurred following nerve activation in these same first order central neurons. Specifically, we computed the change in the cumulative probability of evoking a spike as a function of latency following individual stimulation pulses (see Methods). Figure 3A shows the results for our example type I VO neuron. Initially, prior to nerve activation, test pulses evoked spikes in this neuron at monosynaptic latencies (gray trace; 0.7–1.3 ms). Similarly, following each activation block, we found that action potentials were evoked by stimulation of this neuron and remained monosynaptically phase-locked to individual pulses. Importantly however, the probability of spiking was markedly attenuated (Fig. 3A). Notably, each subsequent activation block induced progressively greater levels of reduction at this monosynaptic time scale (Fig. 3A compare blue, red versus green traces, respectively). The significance of this finding was confirmed by quantifying the same relationship across our population of type I VO neurons; overall we observed a nearly 50% reduction of the monosynaptic response after the final activation block (Fig. 3B–D). In comparison, repeated activation of the vestibular nerve did not alter the probability of vestibular afferents to fire action potentials. Figure 3E illustrates the time course of the response of an example vestibular afferent before and after each activation block. This afferent was typical in that it spiked almost instantaneously (200–500 μs) following the initiation of each pulse, and this immediate and robust firing remained constant following each activation block (Fig. 3E). Similar results were obtained across our population of afferents (Fig. 3F). Overall, the probability of spiking before activation was comparable to that measured after each activation block (Fig. 3F–H; P > 0.05). Thus, taken together, these results suggest that activation of the vestibular nerve induces a reduction in the efficacy of the afferent-type I VO neuron synapse of the direct vestibulo-spinal circuitry.

### Neuronal plasticity alters evoked head movements

**Figure 4:**
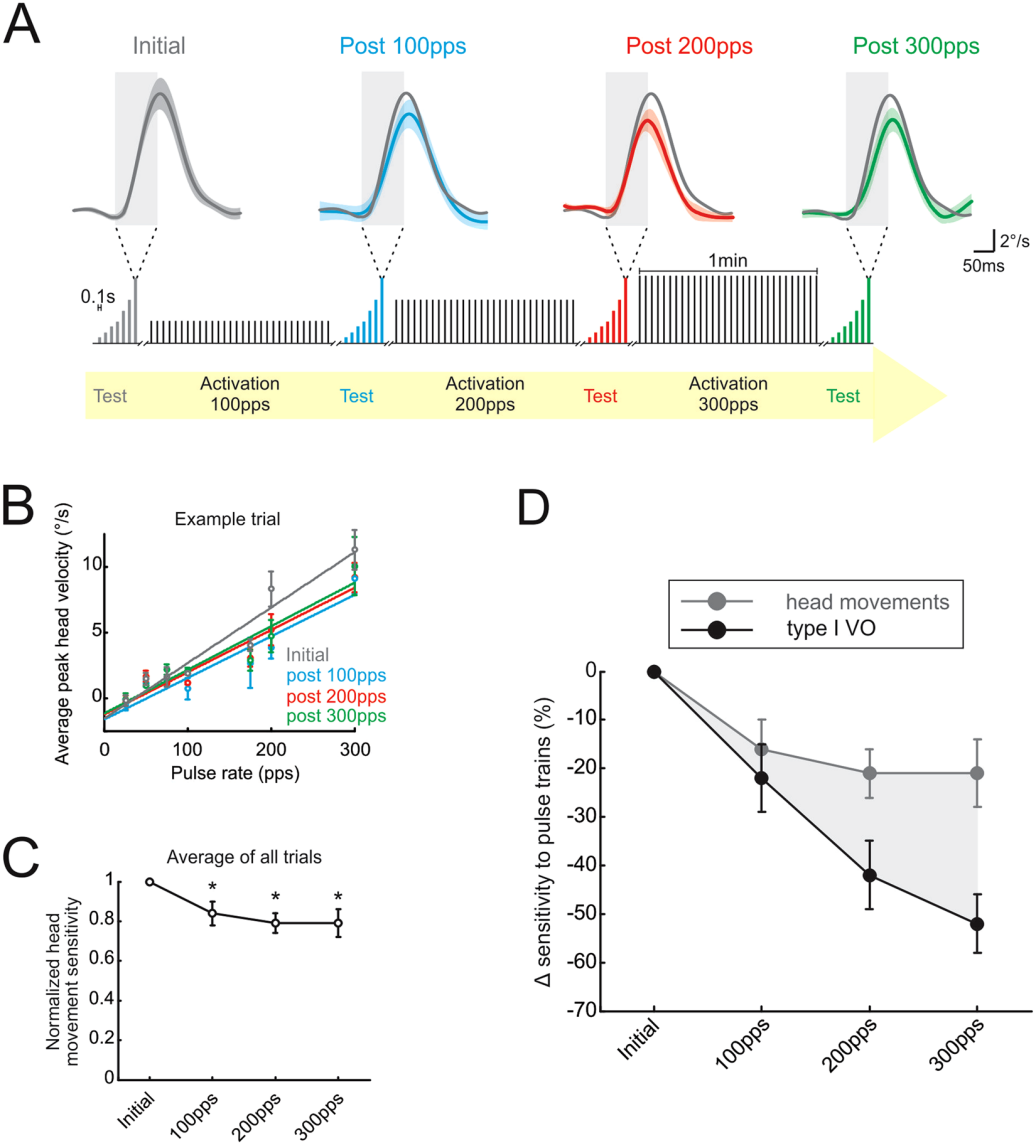
Activation of vestibular nerve decreases magnitude of vestibularly-driven head movements. (**A**) Head movements evoked during test pulse trains of 300pps before and after activation of the vestibular nerve. Inset below shows timeline of stimulation protocol. (**B**) Peak head velocity versus pulse rate during test stimuli before and after activation of the vestibular nerve. (**C**) Head movement sensitivity decreases following activation of the vestibular nerve. (**D**) Comparison between attenuation in head movement and neural sensitivity to test stimuli for type I VO neurons. Error bars and shaded area represent ±s.e.m.

Type I VO neurons send bilateral projections to the cervical segments of the spinal cord (Fig. 1A), and are thus thought to contribute to the vestibulo-collic reflex, which serves to stabilize the head relative to space (reviewed in ref. 2). Accordingly, since stimulation of the vestibular nerve reduces the efficacy of the first central stage of this reflex pathway, we predicted there should be a concurrent decrease in the evoked head movements. To test this hypothesis, we recorded the head movements evoked by stimulation of afferents before and after activation of the vestibular nerve (Fig. 4A inset) in complete darkness. Figure 4A shows example head movements evoked by delivering a short (100 ms) pulse train stimulus at 300pps. Consistent with previous reports^3, 4^, head movements were initially evoked at latencies of ~40 ms (gray traces in Fig. 4A). Furthermore, while latencies were comparable following nerve stimulation, peak head velocity decreased from ~11 to 9°/s (Fig. 4A; compare blue, red, green to gray traces).

To quantify this result, we analyzed the head movements evoked over the full range of pulse rates applied during our test stimuli (25–300pps; Fig. 4B). Specifically, head movement sensitivity to the test sequence stimulation was calculated as the slope of the regression between pulse rate and peak head velocity. Average peak head velocities initially increased with pulse rate (gray data points in Fig. 4B), and the slope of this relationship was reduced following activation of the vestibular nerve at 100pps (blue data points in Fig. 4B). Head movement sensitivity was also similarly reduced following activation at 200 and 300pps (red and green data points in Fig. 4B). Overall, the attenuation of head movement amplitude following vestibular nerve activation was significant relative to baseline values (P < 0.05; Fig. 4C). Thus these findings confirm our original hypothesis that a reduction in the efficacy of the vestibular afferent-type I VO neuron synapse translates into a decreased postural response mediated by the vestibulo-spinal circuitry.

If the relationship between type I VO neuron firing and the magnitude of the vestibulo-collic reflex is roughly linear, then there should be a correspondence between the percent attenuation of neuronal and head motion responses observed following vestibular nerve activation. However, direct comparison of the percent change in type I VO neuron and head movement sensitivity revealed a reduction in neuronal responses that was much greater than the reduction in head movements (Fig. 4D). This difference became particularly marked following the activation block during which 300pps stimulation was applied. Overall, the sensitivity of type I VO neurons was attenuated by 50% whereas the sensitivity of evoked head movement was only attenuated by ~20%. Given the discrepancy between % attenuation observed for neuronal versus behavioral responses, we hypothesized that rapid plasticity at other sites within the vestibulo-spinal reflex circuitry compensated for the reduced efficacy of the afferent-type I VO neuron synapse.

### Rapid plasticity in indirect inhibitory brainstem pathways

**Figure 5:**
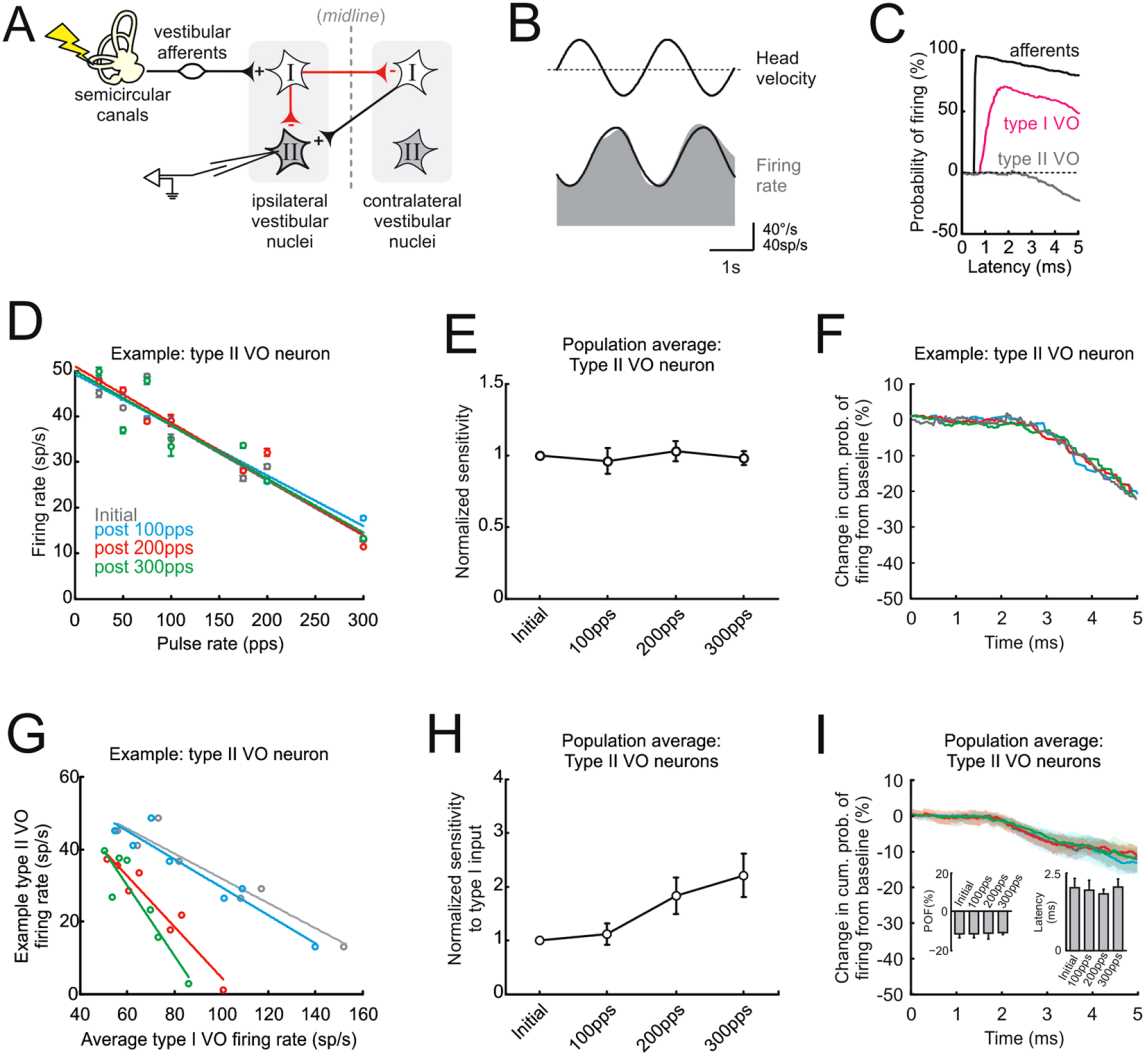
Inhibitory vestibular nuclei pathways display rapid compensatory plasticity. (**A**) Diagram of possible inhibitory pathways linking ipsilateral vestibular nerve and type II VO neurons. (**B**) Example type II VO neuron increases its firing as a function of contralateral head velocity. (**C**) While vestibular afferents are activated almost immediately and type I VO neurons at monosynaptic latencies, type II VO neurons show a decrease in probability of firing with a latency consistent with their response being mediated by polysynaptic pathways. (**D**) Firing rate as a function of pulse rate for example type II neuron before and after activation of the vestibular nerve. (**E**) Normalized sensitivity for the population of type II VO neurons. (**F**) Change in cumulative probability of firing from baseline as a function of time from onset of individual pulses for example neuron. (**G**) Firing rate of example type II VO neuron as a function of averaged type I VO neuron firing rate. (**H**) Normalized sensitivity of type II VO neurons relative to type I VO neuron input. (**I**) Change in cumulative probability of firing from baseline as a function of time from onset of individual pulses for the population of type II neurons. Insets show probability of firing (POF) and latency calculated from the change in probability of firing from baseline curves for type II VO neurons. Error bars and shaded area represent ±s.e.m.

Inhibitory commissural connections between the vestibular nuclei are known to play an important role in the compensation process following peripheral vestibular loss, and long-term changes in these commissural pathways are commonly thought to have an important role in facilitating postural recovery (reviewed in ref. 32). If changes also occur in these indirect inhibitory pathways over the more rapid time scale described above for direct pathway neurons, then complementary plasticity within inhibitory vestibular nuclei pathways could serve to offset this reduction in the efficacy and to improve sensorimotor performance. To directly test this proposal, we next recorded the single unit activity of type II VO neurons. As illustrated in Fig. 5A, these neurons receive ipsilateral vestibular nerve input indirectly through inhibitory pathways^24, 28, 33, 34^. Figure 5B shows the firing rate modulation of an example neuron during passive whole body rotation. Notably, this neuron was typical in that its firing rate increased during contralateral head motion (i.e., type II response) rather than during ipsilateral head motion as for the type I VO neurons described above. On average, type II VO neurons were modulated with a sensitivity of 0.59 ± 0.12 (sp/s)/(°/s) and responses led head velocity by 25 ± 9°. Moreover, consistent with their connectivity, type II VO neurons were not activated by nerve stimulation at monosynaptic latencies. Instead the probability of their firing decreased following nerve stimulation and this decrease occurred at significantly longer latencies (2.5 ± 0.2 ms), consistent with the disynaptic and polysynaptic inhibitory pathways illustrated in Fig. 5A. Figure 5C shows the change in the probability of firing from baseline as a function of latency from the onset of individual pulses for the same example type II neuron, as well as a typical vestibular afferent and type I VO neuron for comparison.

To determine whether rapid complementary changes occur in these indirect pathway neurons following repeated activation of the vestibular nerve, we recorded neuronal activity during a short test sequence (25–300pps) before and after each of the three activation blocks (i.e., 100, 200 and 300pps) using the same approach described above for type I VO neurons. The response of an example cell is shown in Fig. 5D. Notably in the baseline condition (gray data points), this neuron’s response decreased with increasing pulse rate – in striking contrast to type I VO neurons (compare Figs 2E and 5D, respectively). To quantify this effect, we again computed the slope of the linear regression relating pulse rate and firing rate. Interestingly, following activation of the vestibular nerve at 100, 200 and even 300pps, the neuron’’s sensitivity did not change (i.e., slopes remained constant across conditions in Fig. 5D). This example neuron was typical of our population of type II VO neurons (N = 15); overall neuronal sensitivities remained unchanged before and after activation of the vestibular nerve (P > 0.05; Fig. 5E).

The finding that the sensitivity of neurons within local inhibitory pathways were unaffected by activation of the vestibular nerve is noteworthy given that the input to these neurons during our unilateral stimulation protocol was provided by type I vestibular nuclei neurons whose sensitivities did decrease (Fig. 5A). To better understand the implications of this result, we evaluated whether our findings held over a shorter time scale. Specifically, we computed the change in the probability of evoking a spike from baseline as a function of latency following individual pulses before and after nerve activation (Fig. 5F,I, see Methods). As expected, the probability of firing decreased from baseline following individual pulses with latencies consistent with the disynaptic/trisynaptic connectivity of these neurons to the vestibular nerve being stimulated across all conditions (Fig. 5F–I). In addition, the probability of a spike occurring following stimulation remained constant after vestibular nerve activation block (left inset Fig. 5I) with a fixed latency (right inset Fig. 5I), thereby establishing that indeed our findings held over this shorter time scale.

Taken together these results show that the sensitivities of type II VO neurons do not change in response to repeated activation of the vestibular nerve. As noted above, this finding is rather surprising because their input during our applied unilateral stimulation was from monosynaptically-driven type I neurons, whose sensitivities showed a significant decrease in their responses following activation (Figs 3 and 5A). To further explore the implications of this result, we plotted the input-output relationship between type I and type II neurons (gray data points in Fig. 5G). Note that the slope of this relationship effectively provides an estimate of the sensitivity of the local inhibitory pathways relative to the available type I signal. In this context, it is clear that for a given type I input, type II neurons become more sensitive (i.e., show greater inhibition) following repetitive activation of the vestibular nerve (e.g., compare gray and green data points in Fig. 5G). Thus, the modulation of neurons within inhibitory brainstem pathways is actually amplified (i.e., augmented inhibition) relative to their input following vestibular nerve activation (Fig. 5H). Taken together, these results are consistent with our prediction that local inhibitory pathways in the vestibular nuclei compensate for the decreased response of the type I VO neurons, thereby allowing vestibulo-spinal pathways to maintain a relatively robust behavioral output.

### The time course of neuronal responses and motor performance

**Figure 6:**
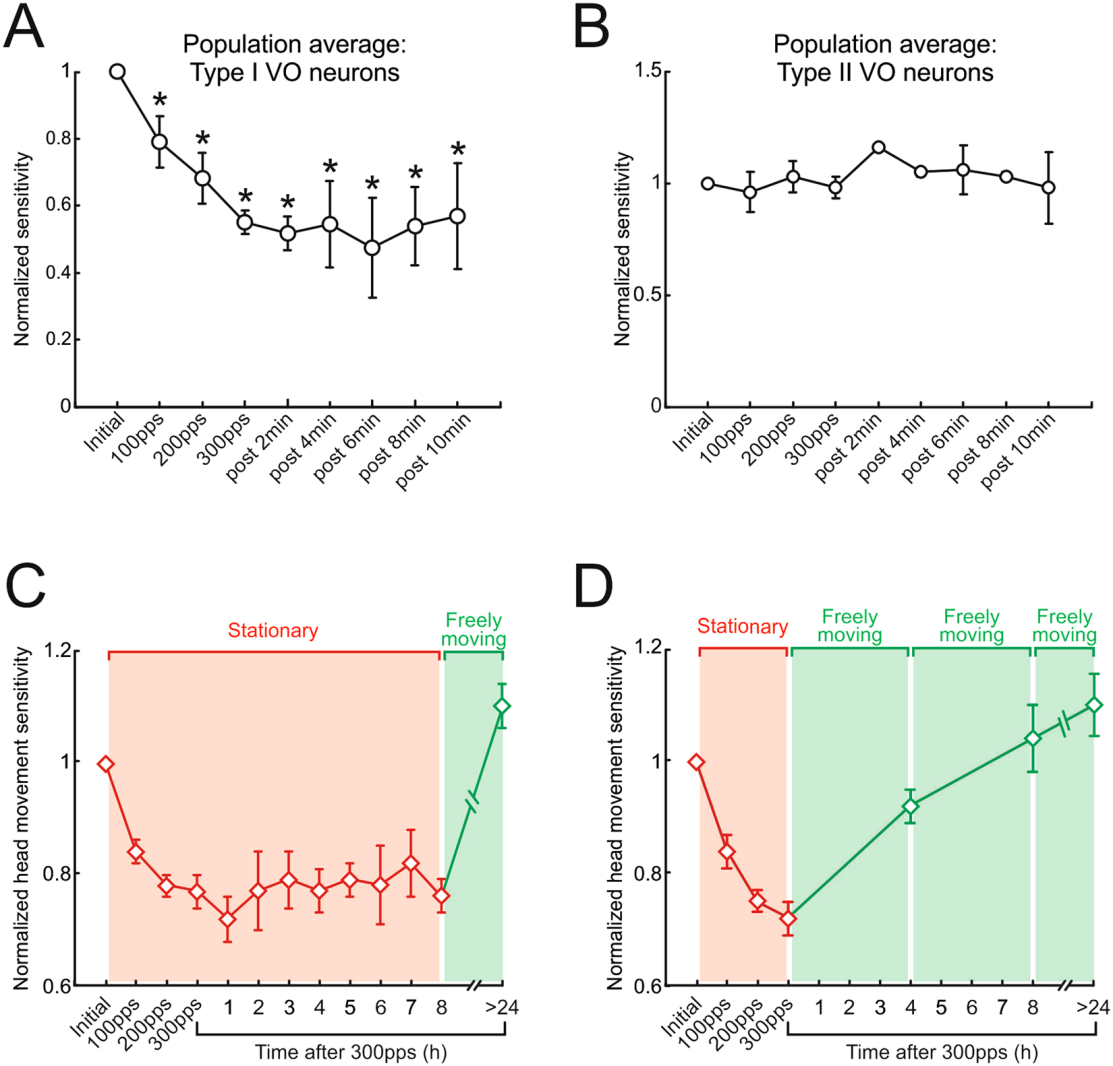
Recovery of neuronal and behavioral responses following activation of the vestibular nerve. (**A,B**) Normalized sensitivity over a period of 10 minutes for population of type I (**A**) and type II (**B**) VO neurons following activation of the vestibular nerve. (**C**) Normalized head movement sensitivity for an 8-h period following activation of the vestibular nerve when animal remained stationary. (**D**) Normalized head movement sensitivity for an 8-h period following activation of the vestibular nerve when animal returned to its home cage in between testing sessions. Error bars represent ±s.e.m.

Thus far, we have shown that repeated activation of the vestibular nerve *in vivo* induces plasticity at the first central stage of vestibulo-spinal pathways that in turn causes a reduction in the induced behavioral response. Specifically, using an approach similar to that applied in previous *in vitro* studies using mouse slice preparation, we found that a reduction in synaptic efficacy at the first central stage of the vestibulo-spinal pathway produced a decrease in evoked head motion, thus linking changes in synaptic efficacy and motor performance. Notably, previous *in vitro* studies have shown that long-term depression at the vestibular afferent-central neurons synapse can persist for up to 30 minutes^24, 25^. Accordingly, to assess the persistence of the attenuation induced in our *in vivo* experiments, we applied our short test sequence every 2 minutes for up to 10 minutes following the last nerve activation block at 300pps in complete darkness. We then quantified the sensitivities of both type I and II VO neurons as described above, based on the slope of the linear regression relating pulse rate and firing rate. As can be seen in Fig. 6A, the efficacy of type I VO neurons was substantially reduced immediately following the last activation block, and even after 10 minutes, neuronal responses showed little recovery and remained significantly attenuated relative to initial values (~40%, p < 0.05). The responses of type II VO neurons, on the other hand, were unchanged following the last activation block relative to initial values, and then remained constant and robust throughout the course of this experiment (Fig. 6B).

*In vitro* studies have also shown that long-term depression (LTD) at other synapses in the brain can last for hours and even days^35, 36^. Although it was not possible to record neuronal activity for this length of time with the techniques employed in this study, we were able to monitor the head movement behavior for up to 8 hours following vestibular nerve activation as a readout of the plasticity within vestibulo-spinal pathways. Remarkably, we found that our brief repetitive stimulation of the vestibular nerve induced a lasting decrease in evoked vestibulo-spinal response. Notably, the evoked head movement remained significantly attenuated for an 8-hour period, when the animal was kept stationary in the dark between testing sessions (Fig. 6C). We further found that when we tested behavioral responses the following morning, they had recovered to baseline values (Fig. 6C). This lead us to hypothesize that the animal’s active motion, generated while freely moving in its home cage overnight, might help induce recovery. To test this possibility, we completed a final experiment in which we again monitored the head movement behavior for up to 8 hours following vestibular nerve activation (as in Fig. 6C) but this time returned the animal to its home cage, where it moved around freely, between each test session. In this condition, we found that behavioral responses recovered more quickly, showing significant improvements in as little as 4 hours (Fig. 6D). Taken together, these results suggest that natural active motion can expedite recovery in vestibulo-spinal pathways when the synaptic efficacy of the afferent-central neuron synapse is attenuated by repetitive activation of the vestibular nerve.

## Discussion

In this study, our goal was to investigate the mechanisms underlying plasticity at different sites within the vestibulo-spinal circuitry of monkeys using temporally precise activation of vestibular afferents combined with single-unit recordings. We found that the probability of evoking a spike in neurons receiving direct vestibular afferent input was significantly attenuated, on a monosynaptic timescale, following behaviorally relevant rates of vestibular nerve activation. In contrast, the timing and amplitude of the probability of spiking in vestibular afferents remained constant indicating nerve activation specifically reduced the efficacy of the vestibular afferent-central neuron synapse. This same stimulation also caused a coincident decrease in motor performance, although the reduction was less than that observed in the direct vestibulo-spinal pathway. Recordings from neurons within indirect brainstem pathways provided insight into the mechanism underlying the difference between the reduction in neuronal activity and motor performance. Notably, the probability of spiking for ipsilateral neurons within local inhibitory pathways remained unchanged following activation of the vestibular nerve. This result was surprising, since, during our unilateral stimulation protocol, the origin of the input to these neurons was first-order central neurons, whose responses did decrease. Thus, taken together, our findings strongly suggest that rapid complementary changes within local inhibitory pathways compensate for decreases in efficacy of the direct vestibulo-spinal pathway, thereby ensuring the maintenance of a robust motor output.

Following behaviorally-relevant rates of vestibular nerve activation *in vivo*, we observed within minutes a reduction in the efficacy of transmission between vestibular afferents and their central targets in the vestibular nuclei. This result complements those of previous *in vitro* studies, which found that comparable nerve stimulation induces long-term depression (LTD) at the vestibular afferent-vestibular nuclei neuron synapse^24, 25^, suggesting that LTD at this synapse also contributes to plasticity *in vivo*. Another potential explanation for the reduced efficacy of the afferent-vestibular nuclei synapse is that inputs from other areas known to project to the vestibular nuclei - regions of the cerebellum such as the anterior vermis, nodulus, and ventral uvula^37, 38^ and/or cortex^39–42^ -induce long-term plasticity in vestibular nuclei neurons. For example, modulation of cerebellar circuits induced by vestibular nerve stimulation could cause homeostatic plasticity resulting in dis-facilitation or inhibition of vestibular nuclei neurons^23^. However while inactivation of the vestibular cerebellum can eliminate plasticity in vestibulo-spinal pathways^21^, to date, previous studies have exclusively focused on longer term changes in vestibulo-spinal pathways: changes that occur over hours^20, 21^, days^22, 43^ and weeks^22^. In addition, we found no change in the resting discharge or regularity of type I VO neurons. This result is consistent with those of McElvain *et al*.^24^, suggesting that in the absence of stimulation intrinsic mechanisms contribute to baseline activity. Overall, our present results indicate that neurons at the first central stage of vestibulo-spinal processing^7–10^ demonstrate significant plasticity in response to their sensory afferent input within minutes – a time frame consistent with the LTD that has been found *in vitro*.

Two principal effects of the plasticity induced in our study were (1) a significant and rapid reduction in the response of first-order central neurons, and (2) a prolonged decrease in the behavioral efficacy of the vestibulo-spinal pathways. Strikingly, the time course of both effects mirror those observed in a recent study of the direct VOR pathway in which plasticity was induced using comparable patterns of vestibular nerve activation^44^. Specifically, in awake, behaving monkeys, first-order central vestibular neurons in both vestibulo-spinal and VOR pathways decreased by ~50% following activation of the vestibular nerve. Moreover, both the vestibulo-spinal reflex and VOR behavioral responses were attenuated, however, the relative decreases were less than the respective changes in the neurons comprising direct reflex pathways. In addition, a third effect of this plasticity, common between pathways, was the invariant nature of neural responses within indirect pathways; the modulation of type II neurons remained robust and unchanged following the induction of plasticity. We speculate that these neurons likely correspond to GABAergic neurons described previously^24, 33, 34, 45^ that do not project outside of the vestibular nuclei but instead provide local and commissural inhibition.

A subject’s perception of self-motion can be diminished in response to repeated or prolonged vestibular stimulation – an effect termed vestibular habituation. After experiencing repeated steps of applied head velocity, subjects report an attenuated sense of self-motion that persists over a time course spanning days^46, 47^, and sometimes months^48, 49^. Similarly, vestibularly-evoked behavioral responses can demonstrate habituation. For instance, Kato and colleagues^50^ found that repetitive vestibular stimulation alters performance during a stepping task in a manner consistent with the suppression of vestibulo-spinal reflex responses – an effect that persisted even 1 month after training. In addition, vestibulo-spinal responses can be reduced following repeated exposure to galvanic vestibular stimulation^51^, with complete recovery only occurring after ~2 months. It is notable that the present study focused on the attenuation that occurs in the vestibulo-spinal pathway responses over a much shorter time frame – specifically within the first minutes of applied stimulation. Nevertheless, it is possible that our results provide some insight into the neural mechanisms that contribute, at least in part, to the vestibular habituation observed in prior studies. Indeed to date, the neural mechanisms underlying vestibular habituation are poorly understood; previous studies have found decreased responses within the vestibular nuclei^52–54^, yet did not identify neuronal cell types and it was therefore not possible to link circuit-level changes in vestibular pathways to changes in behavioral responses.

Previous studies have shown that irregular afferents are more sensitive to electrical stimulation than regular afferents^55^. It has been hypothesized that irregular afferents preferentially contribute to vestibulo-spinal reflexes, while regular afferents preferentially contribute to the VOR^56, 57^. Thus, it would be tempting to speculate that plasticity was preferentially induced in vestibulo-spinal pathways. However, we found no differences in the probability of evoking a spike across afferent types. Indeed, this is consistent with our previous work demonstrating plasticity in the direct VOR pathway^44^. Further, it is important to note that a fundamental difference between the vestibular input experienced in everyday life and our experiments is that, in the latter case, vestibular afferent spiking activity was synchronized since each pulse of the applied stimulation train reliably (and nearly simultaneously) produces an action potential in individual vestibular afferents (see Fig. 3E,F). In contrast, during actual applied head movements, vestibular afferents show little to no synchrony^58, 59^. Our analysis of motor performance further revealed faster recovery dynamics when monkeys made voluntary movements rather than remained stationary (Fig. 6C,D), suggesting that the recovery of pathway efficacy is facilitated by extra-vestibular (i.e., proprioceptive and motor efference copy) as well as vestibular signals. Indeed, the up-weighting of extra-vestibular inputs has been linked to faster and more substantial recovery at the level of single neurons within both direct and indirect vestibulo-spinal pathways following peripheral vestibular loss^60^. This likely reflects a form of homeostatic plasticity (reviewed in refs 61–63) that dynamically adjusts synaptic strength via changes in local circuits^64^ to maintain neuronal activity levels and ensure stable circuit function. While excitatory input has been shown to regulate inhibitory circuit tone over a time course of days to weeks in studies of other neural circuits *in vitro* ^65, 66^, our current findings demonstrate compensatory changes within a shorter time frame of seconds to minutes. Persistent network asymmetries in vestibulo-spinal pathways can be induced during development by circuit perturbations (i.e., removing the input from one labyrinth), in turn producing long term postural deformations^67, 68^. We speculate that up-weighting inhibitory pathways ultimately serves as a protective mechanism that stabilizes activity to ensure balanced posture and reflex behavior.

Vestibular nuclei neurons send both ipsilateral and contralateral projections to neck motoneurons in the spinal cord^2, 69^. Given that our results indicate an increase in the efficacy of local (commissural) inhibitory vestibular nuclei pathways, we speculate that type I VO neurons contralateral to the stimulated nerve are more inhibited following high frequency stimulation - effectively reducing their inhibition onto neck motoneurons and resulting in a larger head movement. Further there are additional mechanisms that could contribute to the discrepancy between the attenuation of neuronal and behavioural responses. First, vestibular nuclei neurons receive disynaptic inhibitory input from the vestibular nerve via inhibitory interneurons, which has been shown to impact how they integrate their direct afferent input^70^. Notably, if depression occurs at the afferent to inhibitory neuron synapse, then this would release the inhibition onto type I neurons and diminish the amount of depression that we observed. Second, another class of vestibular nuclei neurons (termed ipsilateral eye and head velocity neurons) also send direct projections down to the spinal cord^27^. We have previously shown that unlike type I VO neurons, the responses of these neurons are unaffected by high frequency stimulation^44^, and hypothesize that this is in part because they receive significant cerebellar as well as vestibular nerve input. Finally, we note that prior studies have shown non-linearities in vestibulo-spinal reflex dynamics such that there may not be a direct scaling between the vestibular nuclei input and motor response^71, 72^.

In summary, here we found in alert, behaving monkeys that significant plasticity occurs at the first central stage of processing in vestibulo-spinal pathways within minutes of altered sensory input – a time frame consistent with the LTD that has been reported *in vitro*. In addition, as noted above, complementary changes in vestibulo-cerebellar pathways may contribute to the plasticity that we observed. Indeed, a recent study has established that the cerebellum rapidly adapts its descending commands in the face of changes in sensory input^73^. Trial-by-trial analysis of the activity of neurons in the rostral fastigial nucleus, a major output target of the cerebellar cortex^74^, revealed that neuronal responses during motor learning were updated when the relationship between the motor command and resultant head motion was altered^73^. These neurons, in turn, send strong projections to the vestibular nuclei, reticular formation, and spinal cord^74–77^. This suggest that rapid plasticity at multiple sites can combine to shape vestibulo-spinal reflexes in order to ensure robust and calibrated motor performance. In this context, our work has important implications to advance the development of vestibular prostheses to treat patients suffering from vestibular sensory loss^78–82^. Prosthetic stimulation can evoke compensatory postural responses^4, 83^, and our findings indicate that complementary enhancement of local inhibitory pathways within the vestibular nuclei contributes to ensuring relatively robust behavioral performance. We further speculate that the development of novel prosthetic stimulation protocols which better replicate variability in afferent activation induced by natural motion will significantly improve patient outcomes.

## Methods

All procedures were approved by both the McGill University Animal Care Committee and the Johns Hopkins Animal Care and Use Committee, in addition to adhering to the guidelines of the Canadian Council on Animal Care and the National Institutes of Health.

### Surgical procedures

Three male rhesus monkeys (M0603163RhO, M060323RhJ and M0608155RhY) were prepared for extracellular recording experiments using aseptic surgical techniques. The animals were chronically implanted with a post to immobilize the head, recording chambers, and scleral search coils as described elsewhere^44^. A stimulating electrode array was implanted using procedures similar to those previously described^44^. Briefly, a mastoidectomy was performed in the left ear and two small holes were made where the ampullae of the superior and horizontal semicircular canals meet, into which a forked electrode array was inserted^84^. An additional hole was made in the posterior semicircular canal adjacent to its connection with the ampulla, into which a single electrode array was inserted. Lastly, one reference electrode was implanted into the common crus of the labyrinth, and another was positioned in extracranial musculature. Throughout the procedure, care was taken to not damage the membranous labyrinth or suction perilymph^85^. Following electrode implantation, monkeys did not exhibit nystagmus towards the implanted side. There was, however, a small asymmetry in the VOR that was only observed during transient high frequency head rotations.

### Data acquisition

During the recording sessions, monkeys were comfortably seated in a primate chair, which was set upon a vestibular turntable. The extracellular single-unit activity of vestibular-only (VO) neurons, located in the vestibular nuclei, was recorded using an enamel-insulated tungsten microelectrode (7–10 MΩ impedance; Frederick Haer Co., Bowdoinham, ME) as previously described^30, 86^. The location of the vestibular nucleus was determined relative to that of the abducens nucleus, identified by its stereotyped discharge pattern during eye movements^29, 87^. Horizontal and vertical gaze as well as head position (recorded using the magnetic search coil technique), turntable velocity [measured using an angular velocity sensor (Watson Inc.)], and target position were low-pass filtered (250 Hz, analog 8 pole Bessel filter), and sampled at 1 kHz. Unit activity was sampled at 40 kHz. The stimulation artifact was removed using an online adaptive filter (Artifact Zapper, Riverbend Instruments) and offline template deletion as previously described^44^. All signals were saved using a computer-based data acquisition system (Plexon). Each unit was analyzed off-line to ensure proper isolation.

### Experimental paradigms

Neurons were initially recorded during standard head-restrained paradigms to characterize their responses to eye movements and head velocity. Monkeys were trained to follow a laser target, which was projected onto a screen (60 cm in front of the monkey) for a juice reward. When the target was displaced between horizontal positions (±5, 10, 15, 20, 25, and 30°), the monkey made saccadic eye movements to track the target. When the target moved sinusoidally (0.5 Hz, ±40°/s) in the horizontal plane, the monkey made smooth pursuit eye movements. Finally, the neuron’s sensitivity to head velocity was quantified by passively rotating monkeys in the dark about an earth-vertical axis (with a frequency of 0.5 Hz, ±40°/s).

After these basic characterizations, we recorded neuronal activity during the delivery of electrical pulses to electrodes implanted in the horizontal semicircular canal using an isolated pulse stimulator (A-M systems), which was set to deliver symmetric biphasic pulses (150 μs/phase; Fig. 1B) while monkeys were head-fixed in complete darkness, as previously described^44^. Briefly, we first recorded the activity of neurons during a series of test pulse trains (25, 50, 75, 100, 175, 200, 300pps) each lasting 1 s to quantify baseline sensitivities to electrical stimulation of the vestibular nerve. We then applied activation stimuli, which consisted of 30 pulse trains lasting 500 ms and were delivered every 2 s over the course of 1 min at pulse rates of 100pps, 200pps and 300pps (Fig. 1B). Following each activation stimulus pulse train, we delivered test pulse trains to determine whether neuronal sensitivities to stimulation of the vestibular nerve had changed relative to baseline levels. Test stimuli were then delivered every 2 minutes for up to 10 minutes following vestibular nerve activation with 300pps. Typically only one cell was recorded during each experimental session. On 3 occasions, we recorded 2 type I VO neurons within the same session. Note, on each of these days we allowed the monkey to freely move its head after the first recording and then waited at least 1 hour before recording the second neuron.

In order to investigate how vestibular nerve activation influences head movement responses to subsequent vestibular nerve stimulation, we performed a separate set of behavioral experiments. We applied 3 blocks of activation stimuli during which we delivered repetitive stimulation via stimulating electrodes in the horizontal canal with pulse rates of 100, 200 and 300pps while the monkey was head-fixed in the dark. Before and after each block of activation stimuli, we released the animal’s head, allowing him to move freely in the yaw axis (that is, earth-vertical rotation). Head and eye position were monitored online, and electrical stimulation was initiated only if the head and eye were stationary (<1°/s) and centered (±10°) on the body and within the orbit, respectively. To avoid confounding voluntary movement, we applied stimulation for a brief (100 ms) period, and we focused on responses evoked in this initial time window, preceding the time required for the generation of voluntary neck responses and the quick phase component of vestibular nystagmus. Head movements were evoked using a series of pulses trains (100 ms) with rates of 25, 50, 75, 100, 175, 200, 300pps. We compared the recovery of these head movement responses over an 8 hour period following activation of the vestibular nerve when the head remained fixed versus when the monkey was returned to its home cage between testing sessions.

### Analysis of neuronal discharges

Analysis was performed using custom algorithms (Matlab, The MathWorks). Gaze, head, target, and table signals were digitally low-pass filtered at 125 Hz. Eye position was calculated from the difference between gaze and head position signals. Gaze, eye, and head position signals were digitally differentiated to produce velocity signals. Neuronal firing rates were estimated using a Gaussian window (SD of 10 ms)^88^.

In this study, we present data from neurons in the medial vestibular nuclei that are sensitive to yaw rotations but are not modulated during eye movements, VO neurons. We verified that neurons did not exhibit eye movement responses during periods of steady fixation and saccade-free smooth pursuit using multiple regression analysis^30, 86, 89^. A least squares regression analysis was then used to describe each neuron’s response to passive head rotations:

1 $$r=bias+(g\times \dot{H})+(a\times \ddot{H})$$

where *r* is the firing rate, *bias* is a constant equal to the resting neural discharge, *g* and *a* are constant coefficients, and $\dot{H}$ and $\ddot{H}$ are head velocity and head acceleration, respectively. Neuronal sensitivities [*S* in (sp/s)/(°/s)] and phases (*φ* in degrees) relative to head velocity were then computed using the following equations:

2 $$S=\sqrt{{g}^{2}+{(2\pi fa)}^{2}}$$

3 $$\phi ={\tan }^{-1}(\frac{2\pi fa}{g})\times (\frac{180}{\pi })$$

where *f* is the frequency of the sinusoidal rotation^90^. We recorded from a total of 37 VO neurons, which were classified as type I (firing rate increased during ipsiversive head movements; N = 22; N = 9 from monkey J, N = 13 from monkey O) and type II (firing rate increased during contraversive head movements; N = 15; N = 6 from monkey J and N = 9 from monkey O).

Standard linear regression techniques were used to describe the relationships between (1) VO neuron firing rate and pulse rate, and (2) peak head velocity and pulse rate. The precise time course of neuronal responses to vestibular nerve activation was calculated using a method previously described^44, 91^. Briefly, we measured the latency of the first spike following each individual pulse in a train delivered at 25pps. From these latency values, we calculated the probability of a spike occurring as a function of latency from pulse onset. The contribution of spontaneous firing was approximated by overlaying a ‘fake’ pulse train (25pps) on a cell’s resting discharge activity and calculating the spontaneous probability of a spike occurring as a function of latency from pulse onset. This spontaneous probability of firing was then subtracted from the raw probability of firing to obtain the change in probability of firing from baseline. The magnitude of the response was calculated as the difference between the maximum response after stimulation and baseline activity, which was then plotted as a function of time. The latency of the response was defined as the x-intercept of the linear regression of the rise or decrease in the net probability of firing. In addition, the same method was used to calculate the precise time course of individual afferent responses to vestibular nerve activation (N = 7). The regularity of the resting discharge of each neuron was measured by computing its coefficient of variation (CV), $\frac{{\mu }_{ISI}}{{\sigma }_{ISI}}$, where ${\mu }_{ISI}$ and ${\sigma }_{ISI}$ are the mean and standard deviation of the interspike intervals (ISI). Finally, to obtain an estimate of the sensitivity of the local inhibitory pathways relative to the available type I signal, we calculated a linear regression of the firing rate of individual type II VO neurons as a function the average firing rate of type I VO neurons for each pulse rate delivered in our test stimuli. Values are expressed as mean ± SEM and a Student’s t-test was used to determine whether the average of two measured parameters differed significantly from each other.
